# Hepatic Vein and Arterial Vessel Segmentation in Liver Tumor Patients

**DOI:** 10.1155/2022/2303733

**Published:** 2022-09-23

**Authors:** Haopeng Kuang, Zhongwei Yang, Xukun Zhang, Jinpeng Tan, Xiaoying Wang, Lihua Zhang

**Affiliations:** ^1^Academy for Engineering & Technology, Fudan University, Shanghai 200000, China; ^2^Liver Surgery Department, Zhongshan Hospital, Fudan University, Shanghai 200000, China

## Abstract

Preoperative observation of liver status in patients with liver tumors by abdominal Computed Tomography (CT) imaging is one of the essential references for formulating surgical plans. Preoperative vessel segmentation in the patient's liver region has become an increasingly important and challenging problem. Almost all existing methods first segment arterial and venous vessels on CT in the arterial and venous phases, respectively. Then, the two are directly registered to complete the reconstruction of the vascular system, ignoring the displacement and deformation of blood vessels caused by changes in body position and respiration in the two phases. We propose an unsupervised domain-adaptive two-stage vessel segmentation framework for simultaneous fine segmentation of arterial and venous vessels on venous phase CT. Specifically, we first achieve domain adaptation for arterial and venous phase CT using a modified cycle-consistent adversarial network. The newly added discriminator can improve the ability to generate and discriminate tiny blood vessels, making the domain-adaptive network more robust. The second-stage supervised training of arterial vessels was then performed on the translated arterial phase CT. In this process, we propose an orthogonal depth projection loss function to enhance the representation ability of the 3D U-shape segmentation network for the geometric information of the vessel model. The segmented venous vessels were also performed on venous phase CT in the second stage. Finally, we invited professional doctors to annotate arterial and venous vessels on the venous phase CT of the test set. The experimental results show that the segmentation accuracy of arterial and venous vessels on venous phase CT is 0.8454 and 0.8087, respectively. Our proposed framework can simultaneously achieve supervised segmentation of venous vessels and unsupervised segmentation of arterial vessels on venous phase CT. Our approach can be extended to other fields of medical segmentation, such as unsupervised domain adaptive segmentation of liver tumors at different CT phases, to facilitate the development of the community.

## 1. Introduction

The liver has a complex vascular structure, and clarifying the vascular system can improve the accuracy of liver disease analysis, diagnosis, treatment, and surgery [1]. Images acquired at different times, from different viewpoints, or by various sensors can be complementary. Therefore, the accurate fusion of helpful information from Computed Tomography (CT) images of different phases is essential [2]. Especially, in liver vessel segmentation, combining CT images from venous and arterial phases is of great significance for improving organ segmentation and assisting physicians in diagnosis [3]. Due to the different contrast between arterial and venous vessels and surrounding tissues under different phases of CT, the existing annotated data almost only annotate arterial vessels in the arterial phase and venous vessels in the venous phase.

**Figure 1:**
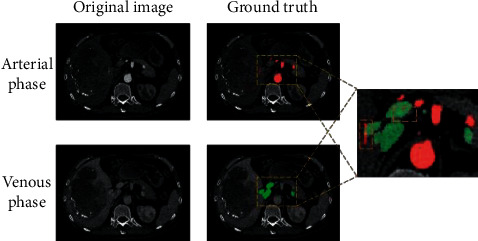
CT images and annotations of the same subject at the same coordinates generated by the same device. The rightmost image is cropped from an arterial phase CT. The red and green areas are the ground truth of arterial and venous vessels, respectively.

Many studies have performed registration to reconstruct the entire vascular system through various means. For example, registration of multiphase data can use clustering and level sets [4], or use fully automated landmark detection and thin plate spline deformation [5]. In addition, a displacement map estimator and a spatial transformer network enable unsupervised learning-based registration [6]. However, these methods cannot accurately reflect the state of arterial and venous vessels simultaneously, which is a significant obstacle to realizing the requirement of real-time reconstruction of the vascular system in modern precision surgery. Specifically, Figure 1 shows the venous and arterial phase image slices of the same subject under the same physical coordinates generated by the same device and the corresponding blood vessel annotation results. We can see that tissues and blood vessels undergo a certain degree of displacement and deformation in different phases. Annotation results of different categories of blood vessels overlap and offset, making it impossible to obtain the entire blood vessel system through simple aggregation directly. Therefore, it is significant to realize the segmentation of the entire vascular system on single-phase CT images. We can ignore the displacement and deformation of blood vessels in different phases and can naturally reflect the state of arterial and venous vessels simultaneously. Based on this, in this paper, we selected CT images of the venous phase as the target to achieve segmentation of arterial and venous vessels.

Segmenting venous vessels on venous phase images is a supervised segmentation problem. Traditional hepatic vessel segmentation algorithms mainly include active contour methods [7–11] that allow adjustment of deformation curves to detect object boundaries, model-based tracking methods [12–16], and least-cost path methods [17, 18]. However, these methods require initialization variables, such as preset initial points or rough segmentation results. They are not enough to accurately segment tiny blood vessels and blurred boundaries. Therefore, with the increase in computing power, the application of deep learning methods that allow extracting the best-fitting internal representation of images for hepatic vessel segmentation is becoming increasingly popular. Examples include portal vein segmentation using CNN [19], hepatic vessel segmentation based on multi-pass CNN architecture [20], attention-guided tandem module [21], and cascaded incremental learning model [22]. Furthermore, since 3D medical images can retain more contextual information, most of the current blood vessel segmentation algorithms are improved based on classic 3D segmentation models, such as 3D U-Net [23], V-Net [24], and densely connected convolutional networks [25]. In recent years, researchers have improved on 3D segmentation model [20, 21, 26–27]. Kitrungrotsakul et al. [20] proposed a multi-path 3D hepatic blood vessel segmentation network VesselNet. Xu et al. [27] proposed a new mean-teacher-assisted confident learning framework. We noticed that the existing supervised deep learning methods for hepatic blood vessel segmentation all focus on the changes to the network structure while ignoring the potential of combining traditional vision-inspired algorithms and deep learning methods in the cross-sectional area.

However, limited by labeling blood vessels, the key to the segmentation task of arterial vessels on venous phase images is to solve the problem of unsupervised domain adaptation. Transfer learning [28] and domain adaptation techniques [29] have been developed to generalize a model trained on the source domain (labeled training dataset) so that it can be applied to the target domain (test dataset). It is worth noting that Generative Adversarial Networks (GANs) [30] provide a way to learn deep representations without extensive annotated training data. GANs are powerful enough to generate high-quality images, and their learning nature is unsupervised. Researchers have made a series of technological breakthroughs around GAN, such as DCGAN [31], CGAN [32], WGAN [33], and CycleGAN [34]. The lack of sufficient public datasets has led to another trend in deep learning methods. Unsupervised deep learning and semi-supervised learning are becoming more common [35–37]. For example, Yao et al. [38] developed an unsupervised domain adaptation framework that significantly improves segmentation accuracy in unlabeled target domains in two challenging cross-modality tasks, namely, brain structure and abdominal multi-organ. Hong et al. [39] proposed a novel unsupervised domain adaptation framework for cross-modal liver segmentation through joint adversarial learning and self-learning. However, most research on unsupervised domain adaptation for image segmentation has focused on simple tasks such as organs and tumors. There is no unsupervised cross-modal research and application for vessel segmentation yet because the size and number of blood vessels observed on CT images of different phases are inconsistent. Another focus of this task is to retain as many vessel features at various levels as possible so that the trained model can obtain better generalization performance.

**Figure 2:**
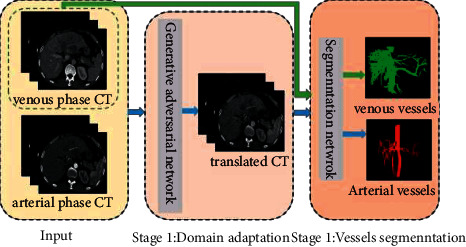
Overall frame diagram. The blue arrows indicate the flow of the unsupervised domain adaptive network for arterial vessel segmentation, and the green arrows mean that the segmentation of venous vessels is supervised.

The data used in this work were all derived from liver tumor patients. Considering that doctors need to observe the relationship between tumors and blood vessels, this study aims to solve how to segment arterial and venous vessels on venous phase CT simultaneously. A two-stage network is designed to achieve it (see Figure 2). The modality conversion from the arterial phase CT image to the venous phase CT image is realized in the first stage. The model trained in the second stage can realize the segmentation of arterial vessels. The segmentation of venous vessels is performed concurrently with the second stage of arterial vessel segmentation. The main contributions of this paper are

1. Unsupervised domain adaptive segmentation of arterial vessels on venous phase CT is realized. Combined with supervised segmentation of venous vessels, we can obtain the complete vascular system on single-phase CT data without registration;
2. We achieved domain adaptation of unpaired arterial and venous phase CT using an improved generative adversarial network. The newly added discriminator strengthens the role of blood vessel information in the network, which can more effectively improve the training efficiency of the network;
3. An orthogonal depth projection loss function is proposed, which uses the 3D geometric information of blood vessels to strengthen the constraints on the segmentation network, further improving the accuracy of blood vessel segmentation.

## 2. Materials and Methods

### 2.1. Data Description

The dataset for this study involved 25 liver tumor patients from Zhongshan Hospital of Fudan University from March 2020 to January 2021. Each subject provided CT images of the venous and arterial phases, corresponding to slice thicknesses of 0.8 or 1 mm. The pixel size of each slice is 512 × 512, the corresponding minimum spacing is 0.619 mm, and the maximum spacing is 0.988 mm. Professional doctors in the hospital provide the ground truth used in training. CT images of 20 subjects were used throughout the experiments for training and validation to select the optimal parameters of the model. The rest of the data are used to evaluate model performance. The environment we use in the experiment is python3.9, torch1.8.1, and Tesla V100-DGXS 32 GB.

Since the original image is a full-abdominal CT image, but the annotations for blood vessels are concentrated in slices with liver tissue, we only extract slices with blood vessel annotations. Furthermore, to reduce the requirement of GPU memory size during training, we compress the size of each slice from 512 × 512 to 352 × 352, which ensures the integrity of the tiny blood vessels. CT images use cubic spline interpolation during the image scaling process, and labels use nearest-neighbor interpolation [40]. By counting the CT values corresponding to the existing tags in the dataset, we set the window width of the CT image to 420 and the window level to 190. After threshold truncating the CT image according to this setting, the effective labeling ratio of blood vessels is 99.08%. Selecting the threshold range can effectively reduce the erroneous labeling caused by unclear blood vessel boundaries. Also, we normalized the data.

**Figure 3:**
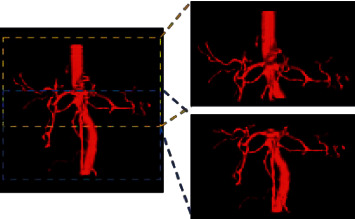
Schematic diagram of selecting 3D patch. The patches are extracted from top to bottom according to the step size of 80 and take at least two patches at the top and bottom of each case.

There are many ways of data augmentation, such as rotation, translation, scaling, and cropping [41, 42]. This study uses random angular rotation to simulate small body twists of subjects during imaging examinations. The simulation of the fatness and thinness of different subjects is achieved by random scaling. In particular, when using 3D models for training, we ensure the consistency of model input by taking 3D patches with dimensions of 96 × 352 × 352 (see Figure 3). This way of cropping data can reduce the demand for GPU, perform data enhancement to a certain extent, and avoid removing more tiny blood vessels by changing the thickness of the slice.

### 2.2. Vascular Reinforcement Domain Adaptive Network

We propose a two-stage model to achieve adaptive vessel segmentation across modality domains. In the first stage, our vascular reinforcement domain adaptive network (VRA-Net) achieves modality conversion from arterial phase CT images to venous phase CT images. The task-oriented new discriminator unit introduces a local information loss function. This design can significantly enhance the representation ability of the generative network for local vascular information.

**Figure 4:**
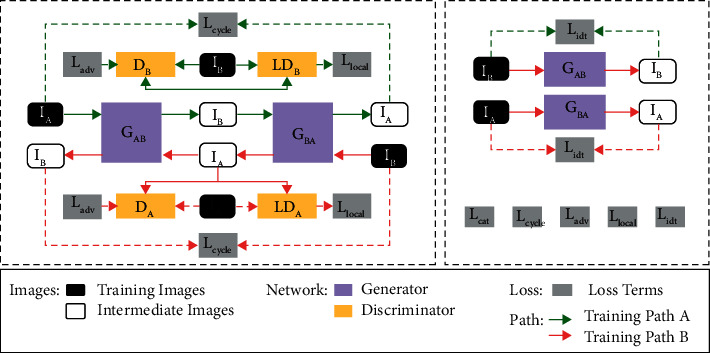
Overall architecture of VRA-NET. The red and green arrows represent the two simultaneous training paths, the solid line represents the network training process, and the dashed line indicates the loss computation.

The VRA-Net is based on the classic CycleGAN [34] with the addition of two new discriminators. As shown in Figure 4, VRA-Net performs adaptive learning between arterial phase CT (domain A) and venous phase CT (domain B). Our VRA-Net has two paths to train simultaneously. One direction is to convert the arterial phase CT image *I*_*A*_ to a pseudo-venous phase CT image $\hat{I}$_*B*_ through the generator *G*_*AB*_. The translated $\hat{I}$_*B*_ then reconstructed into the arterial phase CT image $\dot{I}$_A_ by the generator *G*_*BA*_. Another path starts from *I*_*B*_, generates $\hat{I}$_*A*_ through *G*_*BA*_, and then reconstructs $\dot{I}$_*B*_ through *G*_*AB*_.

**Figure 5:**
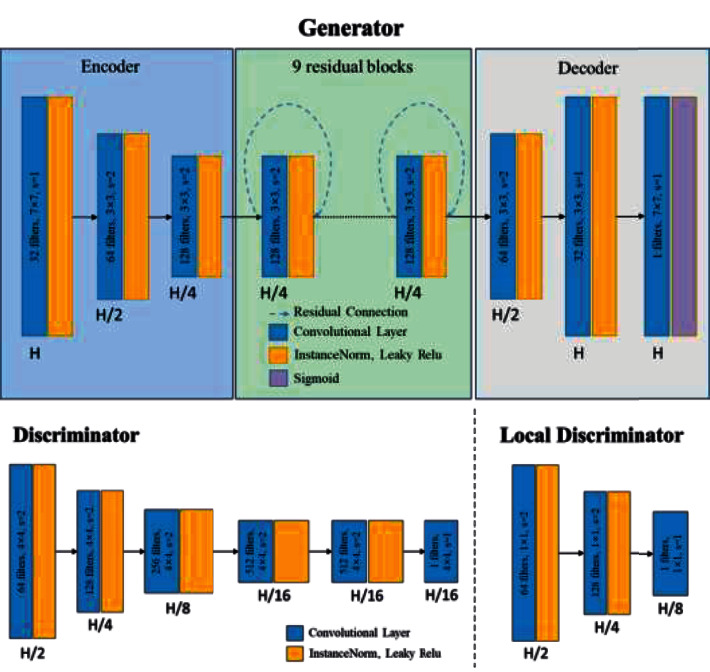
Network structure of generators and discriminators in VRA-NET.

The VRA-Net is a two-dimensional network, which means the network input is 2D slices. VRA-Net contains two generators and four discriminators. The network structures of the generators and the discriminators are shown in Figure 5. The goal of the generator G_AB_ is to convert arterial phase CT images into venous phase CT images. The generator G_BA_ can complete the translation from venous phase CT images to arterial phase CT images. The discriminator *D*_*A*_ and discriminator LD_A_ identify arterial phase CT and pseudo arterial phase CT. Similarly, the discriminators *D*_*B*_ and *LD*_*B*_ identify natural and pseudo venous phase CT.

Traditional adversarial training does not guarantee that tiny blood vessels in an image will look like we want. Some small veins are only a few pixels in size on an image, which is not suitable for multi-layer convolution operations. Unlike *D*_*A*_ and *D*_*B*_, which extract global information from images, *LD*_*A*_ and *LD*_*B*_ only focus on local information related to blood vessels. We use the ground truth *Y*_*B*_ and *Y*_*A*_ of the corresponding blood vessels to activate $\hat{I}$_*B*_ and $\hat{I}$_*A*_, which means only the elements in the corresponding image with annotated positions. Then they are retained as the input of the new discriminator (see Figure 5). This design enables our adversarial training to achieve both global and local style transfer of small blood vessels.

During the training process, the entire network needs to be jointly constrained by four different loss functions.

(1) The classic adversarial loss *L*_adv_ is to make the generated images more like target domain images. The ultimate goal of adversarial training is to make the images generated by the generator indistinguishable from the discriminator. According to the cross-entropy loss [43], the two adversarial losses introduced during network training are
  (1) $$\begin{array}{c} L_{a\;dv}\left(D_{B},I_{B},{\hat{I}}_{B}\right)=E_{I_{B}∼B}\left[\log D_{B}\left(I_{B}\right)\right]+E_{I_{A}∼A}\left[\log\;\left(1-D_{B}\left({\hat{I}}_{B}\right)\right)\right]\text{,}\\ L_{a\;dv}\left(D_{A},I_{A},{\hat{I}}_{A}\right)=E_{I_{A}∼A}\left[\log D_{A}\left(I_{A}\right)\right]+E_{I_{B}∼B}\left[\log\;\left(1-D_{A}\left({\hat{I}}_{A}\right)\right)\right]\text{.} \end{array}$$
(2) The newly added local information loss *L*_local_ ensures that the microvascular information is preserved as much as possible during the domain adaptation process. Similar to *L*_adv_'s definition, we introduce two local information losses:
  (2) $$\begin{array}{c} L_{local}\left({LD}_{B},I_{B},{\hat{I}}_{B},Y_{B}\right)=E_{I_{B}∼B}\left[\log{LD}_{B}\left(I_{B}\right)\right]+E_{I_{A}∼A}\left[\log\;\left(1-{LD}_{B}\left(I_{B}∙Y_{B}\right)\right)\right]\text{,}\\ L_{local}\left(LD_{A},I_{A},{\hat{I}}_{A},Y_{A}\right)=E_{I_{A}∼A}\left[\log{LD}_{A}\left(I_{A}\right)\right]+E_{I_{B}∼B}\left[\log\;\left(1-{LD}_{A}\left({\hat{I}}_{A}∙Y_{A}\right)\right)\right]\text{.} \end{array}$$
(3) The cycle consistency loss *L*_cycle_ is to constrain the source domain image reconstructed by the two generators with the original image to avoid the generator from over-learning the target domain. According to the L1 loss [43], the cycle consistency loss is
  (3) $$\begin{array}{c} L_{cycle}\left(I_{A},{\dot{I}}_{A},I_{B},{\dot{I}}_{B}\right)=E_{I_{A}∼A}\left[{\left|\left|{\dot{I}}_{A}-I_{A}\right|\right|}_{2}^{2}\right]+E_{I_{B}∼B}\left[{\left|\left|{\dot{I}}_{B}-I_{B}\right|\right|}_{2}^{2}\right]. \end{array}$$
(4) The role of the identity loss *L*_idt_ is to ensure that the generated images are not mapped to other domains when the input to the generator is from the target domain. *L*_idt_'s expression:
  (4) $$\begin{array}{c} L_{i\;dt}\left(I_{A},{\hat{I}}_{A},I_{B},{\hat{I}}_{B}\right)=E_{I_{B}∼B}\left[{\left|\left|{\hat{I}}_{B}-I_{B}\right|\right|}_{2}^{2}\right]+E_{I_{A}∼A}\left[{\left|\left|{\hat{I}}_{A}-I_{A}\right|\right|}_{2}^{2}\right]. \end{array}$$
Finally, the overall target loss for VRA-Net is a weighted combination of the above losses:
  (5) $$\begin{array}{c} L_{VRA}=\lambda_{1}L_{cycle}+\lambda_{2}L_{a\;dv}+\lambda_{3}L_{local}+\lambda_{4}L_{i\;dt}. \end{array}$$

### 2.3. Vascular Segmentation Network Constrained by Orthogonal Depth Projection

The modality conversion of arterial and venous phase CT images can be realized using VRA-Net. Combined with the clinical needs of doctors, our goal is to achieve the segmentation of arterial and venous vessels on venous phase images. We only need to combine the pseudo venous phase 2D images generated by the generator into corresponding 3D volume images for each subject and use the related arterial blood vessel labels as the ground truth on the generated dataset. We can use the classical supervised training of the segmentation algorithm.

**Figure 6:**
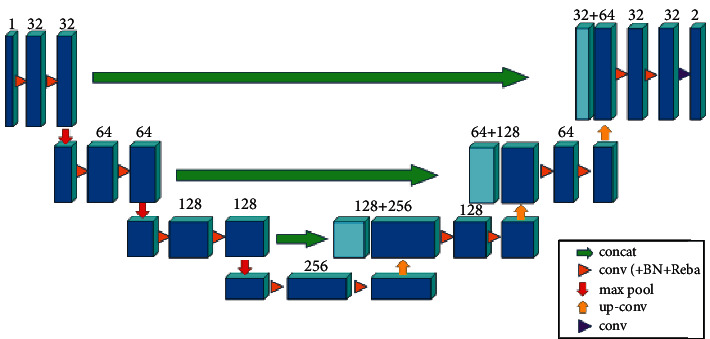
Network structure of vascular segmentation network.

Because there are different levels of branch vessels in the whole vascular system and their directions are different, some small blood vessels only appear in one image and are only a few pixels in size. To make more use of the 3D information of blood vessels, the main body of the segmentation network structure used in this paper is the classic 3D U-Net [23] (see Figure 6 for details). The whole network has four layers of downsampling and four layers of upsampling. Each layer in downsampling consists of two 3 × 3 × 3 convolutions, batch normalization, ReLu [44], and max_pooling. There are two inputs in the upsampling process, one is from the next layer, and the other is the output from the same layer of downsampling. Splicing the two inputs together ensures that the restored feature map incorporates features of different scales.

In addition, in addition to the Dice loss function [45], we propose a novel and effective projection similarity loss to improve the segmentation network's ability to learn the 3D geometric information of the vascular system during training. The core of the projection loss function is to calculate the cosine similarity of the orthogonal depth projection of the 3D model on the *x*, y, and *z* planes. The formulas of the two loss functions are as follows:

(6) $$\begin{array}{c} L_{di\;ce}=1-\frac{2\left|X\cap Y\right|}{\left|X\right|+\left|Y\right|}\text{,} \end{array}$$

(7) $$\begin{array}{c} L_{ps}=1-\frac{1}{3}\left(\frac{P_{x}\left(X\right)∙P_{x}\left(Y\right)}{{\left|P\right.}_{x}\left(X\right)|\times \left|P_{x}\left(Y\right)\right|}+\frac{P_{y}\left(X\right)∙P_{y}\left(Y\right)}{{\left|P\right.}_{y}\left(X\right)|\times \left|P_{y}\left(Y\right)\right|}+\frac{P_{z}\left(X\right)∙P_{z}\left(Y\right)}{{\left|P\right.}_{z}\left(X\right)|\times \left|P_{z}\left(Y\right)\right|}\right), \end{array}$$

where *X* is the element representing the blood vessel in the prediction result, *Y* means the element of the blood vessel in the ground truth, |X∩Y| is the intersection between *X* and *Y*, and |X| and |Y| are the number of elements. In addition, *P*_*x*_, *P*_*y*_, and *P*_*z*_ are orthogonal depth projection functions, and *x*, *y*, and *z* are projection directions. Our projection function is designed for the mask obtained by segmentation during network training, a binary 3D matrix. The specific way to find the orthogonal depth projection is summed along a certain axis (*x*, *y*, or *z*) to obtain the density matrix of the vessel model in that direction and expand it into a one-dimensional vector.

Finally, using the parameter *λ* to control the influence of different losses, the overall target loss *L*_Seg_ of the segmentation network is expressed as

(8) $$\begin{array}{c} L_{Seg}=L_{di\;ce}+\lambda L_{ps}. \end{array}$$

### 2.4. Evaluation Indicators

In this paper, we use a popular metric, Dice Similarity Coefficient (DSC), to evaluate the segmentation results of different organs. The meaning of each symbol is consistent with that in Equation (6). The formula is

(9) $$\begin{array}{c} DSC=\frac{2\left|X\cap Y\right|}{\left|X\right|+\left|Y\right|}. \end{array}$$

## 3. Results and Discussion

### 3.1. Experimental Results of VRA-Net

During the generative adversarial training process in the first stage, we set *λ*_1_ = 10, *λ*_2_ = *λ*_3_ = 1, and *λ*_4_ = 5. After the training, we extracted the generator from the trained model to test the effect of pseudo-venous phase images generated by arterial phase CT. For the effect of modal transformation, we conducted a qualitative comparative study. The difference between our method and the classic CycleGAN is that a new local information loss is introduced through a newly designed discriminator unit.

**Figure 7:**
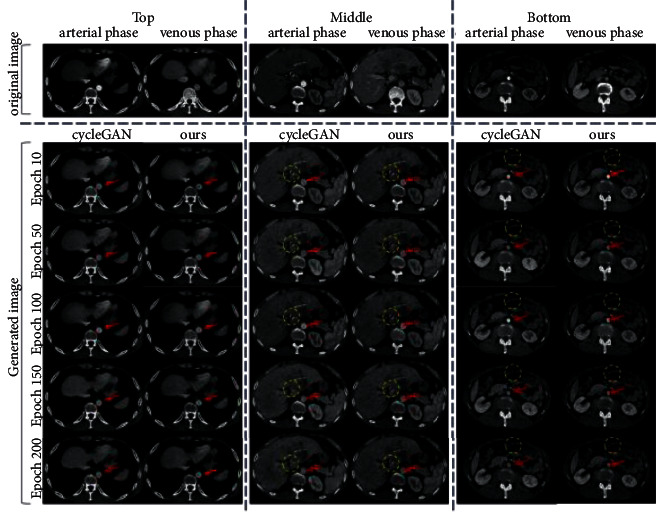
Rendering of the generated pseudo venous phase image. The circular area pointed to by the red arrow is the aortic vessel, and the yellow dotted circle is the representative tiny arterial vessel.

A qualitative comparison of the generated results of domain adaptation is shown in Figure 7. We use test set data that did not participate in any training for testing and select slices from different positions (upper, middle, bottom) in a CT for display. Intuitively, the main differences in arterial vessels between the two phases lie in brightness and contrast with adjacent tissues. From the position indicated by the red arrow, we can observe the changes in the brightness of the arterial vessels in the pseudo-venous phase images generated as the training progresses. The high-contrast location in the yellow circle is the tiny arterial vessel. It can be seen that when the training progresses to 100 epochs, the corresponding positions of the arteries and blood vessels in the generated results of CycleGAN become highlighted again. After that, with the progress of training, although the brightness of arterial blood vessels decreased again, some blood vessels gradually became blurred, some slender branch blood vessels were broken, and some tiny blood vessels even disappeared. Correspondingly, our proposed method solves these problems well.

Experiments show that our introduction of local information loss can improve the generative adversarial network's ability to generate and discriminate tiny blood vessels during domain adaptation, thus making the whole network more robust.

### 3.2. Experimental Results of Vascular Segmentation Network

**Figure 8:**
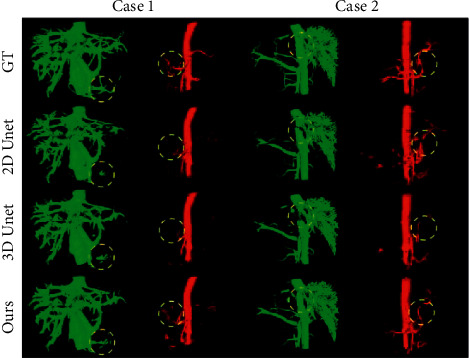
Results of segmentation of arterial and venous vessels on venous phase CT. Representative blood vessels are shown in the yellow dotted circle, and the position shown in the third column is the junction of the inferior vena cava and the liver.

The pseudo-venous phase CT image obtained by the generator is used as the input of the segmentation network, and the corresponding arterial annotation of the original image can be used as the ground truth to supervise the training of the segmentation network. We compare the results of the classical 2D U-Net segmentation network and 3D U-Net and conduct qualitative and quantitative studies. We selected two examples to visualize the segmentation results in qualitative research. We invite doctors to label the arteries and veins on the CT images of the venous phase on the test set not participating in the training. The first row of Figure 8 visualizes the labeling results. As we can observe, the experimental results demonstrate the effectiveness of the vessel segmentation network. After training on modality transformed images, we can achieve arterial vessel segmentation on venous phase images.

In particular, the classical algorithm has poor segmentation results for the junction of the inferior vena cava and the hepatic vein in Case 2. It is not easy to label this junction properly without the doctor's experience, which is surrounded by liver tissue and has poor contrast with the surrounding tissue. This result suggests that the orthogonal depth projection loss can improve the ability of branch vessels to learn geometric information.

We mainly calculated the DSC metrics on the segmentation results in quantitative research. The segmentation results of arterial and venous vessels are shown in Tables 1 and 2. Real A represents the original arterial phase CT image, and fake B represents the venous phase CT image obtained by sending the arterial phase CT image into the generator. Real B represents the original venous phase CT image. In addition, the ground truth used in calculating the arterial segmentation results is the manual annotation results from professional doctors on the corresponding modalities.

From rows 2 and 3 of Table 1, the arterial segmentation model trained on CT in the arterial phase has a poor segmentation effect on arterial vessels in the venous phase. This is obvious because the data distributions are different for the two different modalities. In fact, in this case, the model cannot segment the branches of arterial vessels at all. By comparing lines 1, 3, and 5 or lines 2, 4, and 5, it is not difficult to find that some features of arterial vessels are inevitably lost after modal transformation (domain adaptation). Our method reduces this loss, which further proves that the local information loss function can improve the representation ability of the generator for tiny blood vessels in adversarial training. In addition, the designed projection similarity measure enables the segmentation network to bring about 1.14% and 1.81% performance improvement in arterial segmentation and vein segmentation, respectively.

## 4. Discussion

In this work, we propose a novel task of unsupervised segmentation of blood vessels and establish a general two-stage framework. Specifically, we develop a VRA-Net to adaptively perform modality transfer on arterial phase CT images and venous phase CT images. The generated 2D images are then combined into volume images and fed into the vessel segmentation network to simultaneously complete unsupervised training of the arterial vessel segmentation model and supervised training of the vein segmentation model.

Our research has three important implications. First, our proposed network framework enables unsupervised training of arterial vessels. This scheme, which does not require the target modality ground truth, alleviates the manual annotation requirement for the target modality. On the contrary, our research results can assist in subsequent annotation work. Then, we achieve simultaneous segmentation of arteries and veins in the venous phase, which avoids the need for separate post-segmentation registration work in the arterial and venous phases. In addition, both of our proposed loss functions can enhance the network's ability to represent branch vessels. The effectiveness of the projection similarity measure in the segmentation network shows that the combination of traditional vision-inspired algorithms and deep learning methods has excellent potential in interdisciplinary computer applications.

However, our study still has certain limitations. As seen from the visualization results shown in Figure 8, some tiny blood vessels still cannot be accurately segmented, especially for arterial segmentation, which is unsupervised. This is because our domain-adaptive network focuses on learning domain-invariant features in the two modal data, missing the ability to represent some arterial vessels that are difficult to observe in the venous phase. In addition, our study lacks quantitative assessment methods for tiny vessels. In future research, we can try to segment the liver's interior according to the vascular system's anatomical structure to select the area where some tiny blood vessels are concentrated for quantitative evaluation.

## 5. Conclusions

In summary, we propose a vessel segmentation framework that enables supervised training of venous vessel segmentation and unsupervised training of arterial segmentation on venous phase CT. We demonstrate the effectiveness of this framework. One of the cores of our framework is the unsupervised domain adaptation network, which can greatly reduce the pressure on physicians to annotate data. Our research can be generalized to multi-object segmentation on multi-modal data from multiple centers. The multi-object segmentation achieved in a single phase can naturally avoid the displacement and deformation of organs or tissues caused by data generated at different times. We believe this technology can effectively assist surgical decision-making and even promote the further development of precision medicine soon.

Our study still has some limitations, such as the lack of quantitative assessment methods for tiny vessels and the validation of multicenter data. In future studies, we will further improve the accurate segmentation of tiny vessels and combine the task of liver tumor segmentation to evaluate the possibility of applying this framework to real-time surgery and perform multicenter validation.

## Figures and Tables

**Table 1:** Arterial vessel segmentation results. Real A represents the original arterial phase CT image, and Fake B represents the venous phase CT image obtained by sending the Real A into the VRA-net. Real B represents the original venous phase CT image.

Method	Train data	Test data	Average DSC	Test data	Average DSC
2D U-net	Real A	Real A	0.8741 ± 0.0002	Real B	0.5947 ± 0.0004
3D U-net	Real A	Real A	0.8793 ± 0.0002	Real B	0.6789 ± 0.0032
2D U-net	Fake B	Fake B	0.8315 ± 0.0004	Real B	0.8117 ± 0.0013
3D U-net	Fake B	Fake B	0.8305 ± 0.0006	Real B	0.8340 ± 0.0019
Ours	Fake B	Fake B	0.8505 ± 0.0002	Real B	**0.8454** ± 0.0010

Values in bold indicate that our proposed method achieves the best performance on unsupervised arterial vessel segmentation.

**Table 2:** Venous vessel segmentation results. Real B represents the original venous phase CT image.

Method	Train data	Test data	Average DSC
2D U-net	Real B	Real B	0.7757 ± 0.0010
3D U-net	Real B	Real B	0.7906 ± 0.0022
Ours	Real B	Real B	**0.8087** ± 0.0002

Values in bold indicate the best performance of our proposed method on the task of supervised venous vessel segmentation.

## Data Availability

The data used to support the findings of this study are restricted by the ethics board in order to protect patient privacy. Data are available from the corresponding author for researchers who meet the criteria for access to confidential data.
